# Nutritional Awareness of Extra Virgin Olive Oil: Empirical Evidence from Consumer Perceptions and Health Beliefs

**DOI:** 10.3390/foods15173001

**Published:** 2026-08-26

**Authors:** Manuela Oliverio, Luigi Gencarelli, Martina Michienzi, Sonia Bonacci, Antonio Aquino, Antonio Procopio

**Affiliations:** Department of Health Sciences, Magna-Graecia University of Catanzaro, Viale Europa, 88100 Catanzaro, Italy; m.oliverio@unicz.it (M.O.); luigi.gencarelli@studenti.unicz.it (L.G.); martina.michienzi@libero.it (M.M.); s.bonacci@unicz.it (S.B.); procopio@unicz.it (A.P.)

**Keywords:** extra virgin olive oil, consumer behavior, EFSA health claims, territorial identity, food communication

## Abstract

Extra virgin olive oil (EVOO) is a key component of the Mediterranean diet and a functional food whose health properties are mainly attributed to phenolic compounds. Despite the availability of EFSA-approved health claims, consumers often rely on heuristic cues that may not correspond to scientifically recognized quality indicators. A cross-sectional online survey involving 516 Italian consumers was conducted to investigate consumption patterns, health-related beliefs, territorial identity, and sensory quality perception. Descriptive analyses, regional comparisons, and a multivariable logistic regression model predicting sensory misperception were performed. Most respondents reported frequent EVOO consumption and willingness to pay a premium for certified products. Territorial identity, trust in local producers, and origin-related cues emerged as important dimensions of quality perception. Although respondents generally recognized the health relevance of antioxidants and unsaturated fatty acids, misconceptions regarding sensory indicators were common. Specifically, 41% of respondents associated sweetness with EVOO quality, whereas bitterness and pungency were less frequently recognized. Greater perceived knowledge of EVOO health properties was associated with a lower likelihood of identifying sweetness as an indicator of EVOO quality. Overall, consumer evaluation of EVOO reflected the interaction of informational, symbolic, and experiential dimensions, highlighting the potential relevance of communication strategies and sensory education initiatives whose effectiveness should be evaluated in future research.

## 1. Introduction

**Figure 1:**
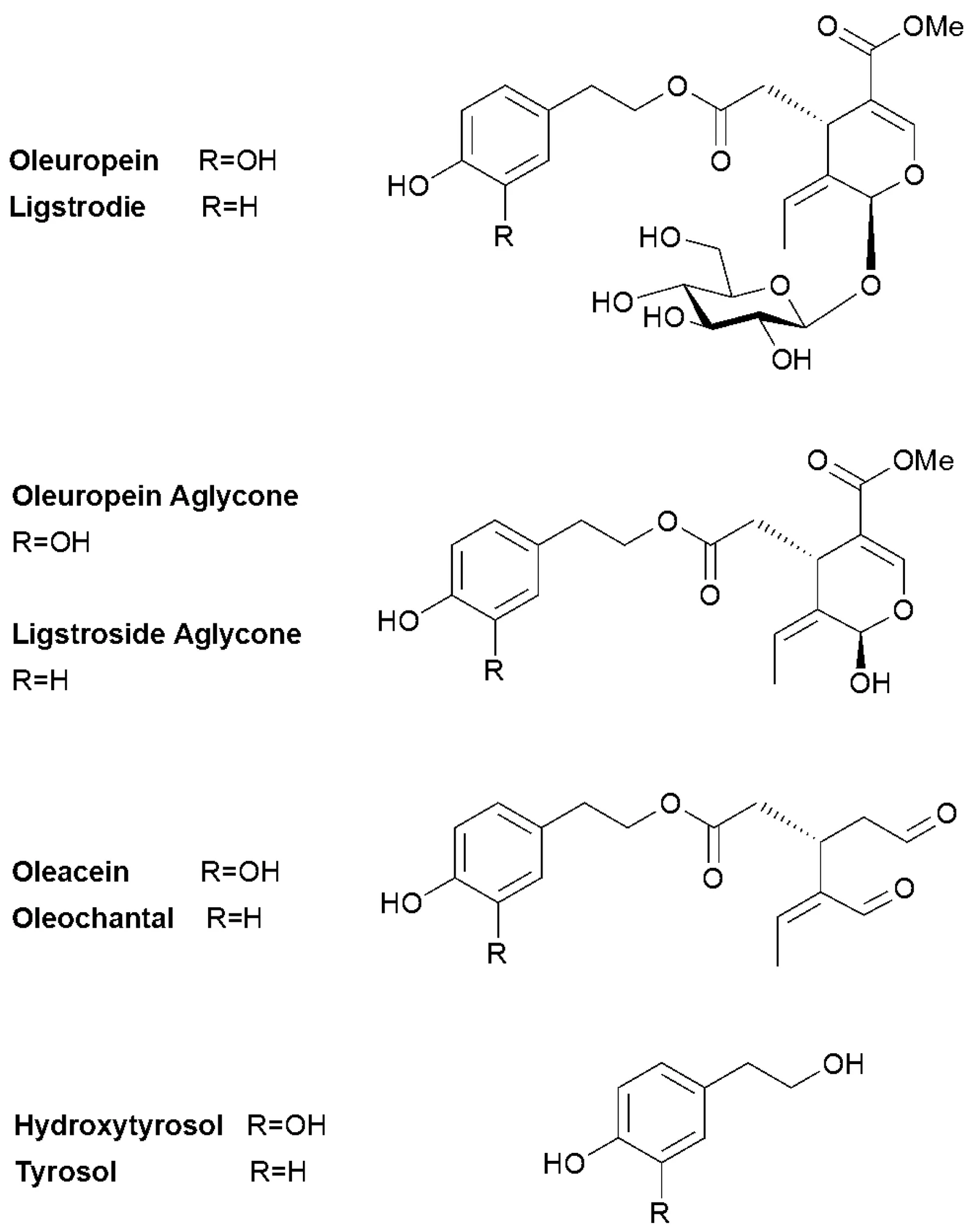
Chemical structures of the main olive secoiridoids and their derivatives associated with the EFSA-approved health claim on olive oil polyphenols.

Extra virgin olive oil (EVOO) represents the primary lipid source of the Mediterranean diet, a dietary pattern associated with reduced morbidity and mortality from chronic, degenerative, and inflammatory diseases [1,2]. EVOO is widely regarded as a functional food whose health-promoting properties derive not only from its high content of monounsaturated fatty acids, particularly oleic acid, but also from its minor bioactive components [3]. Among these, phenolic compounds have received particular attention because of their antioxidant and anti-inflammatory properties and their contribution to both the biological and sensory characteristics of EVOO [3,4]. Secoiridoids constitute a major class of EVOO phenolic compounds and include oleuropein- and ligstroside-derived compounds, such as oleocanthal and oleacin [5,6] (Figure 1).

The biological relevance of EVOO has also received regulatory recognition. Regulation (EU) No. 432/2012 authorizes health claims concerning olive oil polyphenols, oleic acid, vitamin E, and unsaturated fatty acids [7,8,9,10]. These regulatory claims are consistent with the broader evidence concerning the biological activity and health-related properties of EVOO phenolic compounds [11]. In particular, the EFSA-approved claim on olive oil polyphenols states that they contribute to the protection of blood lipids from oxidative damage, provided that the oil contains at least 5 mg of hydroxytyrosol and its derivatives per 20 g of olive oil [7]. These scientifically established health-related properties provide consumers with potentially valuable information for evaluating EVOO. However, the availability of scientifically validated information does not necessarily imply that consumers correctly understand, interpret, or use such information when evaluating product quality.

Beyond their health-related relevance, phenolic compounds also contribute to the sensory characteristics of EVOO [12]. In particular, bitterness is associated with phenolic compounds such as oleuropein derivatives and oleacin, whereas pungency has been linked to oleocanthal [6,13]. These sensory attributes are therefore not merely matters of taste preference, but can provide information about the phenolic profile of the oil and are recognized as positive characteristics in the sensory evaluation of high-quality EVOO [12,14]. Nevertheless, consumers do not necessarily interpret these sensory cues in accordance with their chemical and functional significance. Previous research suggests that consumers may prefer milder, sweeter, or fruitier sensory profiles and may perceive bitterness as undesirable, despite its relationship with phenolic compounds [12,15,16,17,18]. This distinction between sensory preference and the interpretation of a sensory attribute as an indicator of product quality is particularly important. A preference for sweetness does not in itself constitute a misconception; rather, a potential mismatch arises when sweetness is interpreted as evidence of superior EVOO quality, while sensory characteristics more closely associated with phenolic richness, such as bitterness and pungency, are overlooked or negatively evaluated [14,16].

Consumers differ substantially in their ability to interpret health-related information, and knowledge represents a particularly important factor in this process. In the specific context of EVOO, previous research showed that only 36% of Italian consumers adequately understood the meaning of authorized health claims and identified nutrition knowledge as an important determinant of claim understanding [19]. Similarly, individual differences in knowledge about the nutritional and health properties of olive oil have been shown to influence consumers’ perceptions of health-related product attributes [20]. Thus, familiarity with EVOO as a commonly consumed product should not be equated with an accurate understanding of the scientific basis of its health and quality characteristics. This distinction is particularly relevant because consumers are exposed to multiple sources of information when evaluating EVOO. Product labels may simultaneously communicate nutritional information, geographical origin, organic production, certifications, and health-related claims, requiring consumers to select and interpret several potentially competing quality cues. Previous research suggests that consumers may experience difficulties in distinguishing among different types of claims and certifications and that increasing the amount of information available on a label does not necessarily improve its interpretation [21,22]. Consequently, consumer evaluations may depend not only on the information objectively available but also on what consumers know, understand, and perceive themselves to know about EVOO.

Beyond facilitating the understanding of health-related information, consumer knowledge may also shape the broader evaluation and economic valuation of EVOO. Previous studies indicate that education, familiarity with product information, and knowledge can influence consumers’ interpretation of quality cues and their willingness to pay for products carrying specific quality attributes [23,24]. Importantly, recent evidence distinguishes between objective and subjective knowledge, suggesting that these dimensions may play different roles in consumer decision-making and that perceived knowledge can be particularly relevant to willingness to pay for EVOO carrying health claims [24]. At the same time, knowledge does not operate in isolation. Consumers evaluate EVOO through a combination of intrinsic and extrinsic cues, including sensory characteristics, geographical origin, certification schemes, and other information available at the point of purchase [22,25]. Consequently, even consumers who are highly familiar with EVOO may combine scientifically grounded information with experiential expectations and simplified quality cues, as sensory evaluations can also be shaped by prior expectations concerning product characteristics [26]. Understanding consumer evaluation therefore requires considering not only the amount of information available, but also how consumers interpret and integrate that information with their existing knowledge and beliefs.

Beyond knowledge and information processing, EVOO evaluation is also embedded within a broader cultural and territorial context. This dimension is particularly relevant in Italy, where olive oil is closely connected with local production traditions, geographical origin, and regional food identities [25,27]. Previous research has shown that consumers attribute substantial importance to local production, region of origin, and geographical indications when evaluating olive oil quality [25,28]. Regional identity may further shape these evaluations: consumers who identify strongly with an olive-producing territory have been shown to assign greater value to EVOO originating from that same geographical context [27]. Such findings provide a theoretical basis for considering geographical context in EVOO research, as regional variation in consumer perceptions may be associated with differences in familiarity with local production systems, cultural traditions, and the symbolic value attached to olive oil. Territorial context may therefore operate alongside knowledge and sensory experience in shaping how consumers interpret quality-related cues.

Taken together, previous research indicates that consumer perceptions of EVOO quality emerge from the interplay of multiple sources of information [29]. Knowledge may facilitate the interpretation of health-related and compositional information, while sensory experience can shape expectations regarding desirable product characteristics. At the same time, extrinsic cues such as geographical origin, certifications, and territorial identity may provide additional frameworks through which consumers interpret quality [14,16,20,24,25,27,28]. Although previous studies have provided important insights into these different dimensions of EVOO evaluation, their joint consideration may offer a more integrated understanding of how informational, sensory, and territorial factors coexist within consumers’ representations of product quality. This issue is particularly relevant when subjective perceptions diverge from scientifically established product characteristics. Consumers may be highly familiar with EVOO and strongly oriented toward quality while still relying on sensory or informational cues that only partially correspond to recognized indicators of phenolic richness and product quality [14,16]. Examining perceived knowledge together with sensory quality beliefs and territorial cues may therefore provide a more integrated understanding of how consumers construct EVOO quality and of the conditions under which inaccurate quality associations persist.

Against this background, the present study aimed to investigate how Italian consumers understand and evaluate EVOO quality, with particular attention to the interplay between perceived knowledge, information-related cues, sensory beliefs, and territorial context. Rather than considering consumer knowledge solely in terms of familiarity with the product, the study examined perceived knowledge of EVOO health properties together with the informational, sensory, and contextual cues that consumers use when forming quality judgments.

Specifically, the study aimed to: (i) characterize EVOO consumption and purchasing patterns, including willingness to pay for certified products; (ii) examine consumers’ perceived knowledge and health-related beliefs and their relationship with quality-oriented evaluations; (iii) investigate the correspondence between consumers’ sensory preferences and the sensory attributes they interpret as indicators of EVOO quality, with particular attention to the association between sweetness and quality; and (iv) explore whether consumer behaviors and quality perceptions vary across geographical contexts.

Based on previous evidence concerning consumer knowledge, information processing, and sensory evaluation of EVOO, we hypothesized that higher perceived knowledge of EVOO health properties would be associated with a lower likelihood of identifying sweetness as an indicator of EVOO quality (H1). Given the role of geographical origin, local production traditions, and regional identity in EVOO evaluation, we also expected territorial and trust-related cues to contribute to consumers’ broader perceptions of product quality (H2). Finally, we expected that greater attention to quality-related product information would be associated with a greater willingness to pay a premium for certified high-quality EVOO (H3).

## 2. Materials and Methods

### 2.1. Participants, Recruitment and Study Design

A cross-sectional online survey was conducted to investigate consumer behavior, health-related beliefs, and quality perception regarding EVOO. According to institutional and national regulations, ethical approval was not required for this anonymous voluntary survey involving adult participants. Participation was voluntary and anonymous, and respondents were informed about the aims of the study before completing the questionnaire. Electronic informed consent was obtained from all participants prior to participation. No personally identifiable information was collected or stored, and the data were used exclusively for research purposes. The dataset generated and analyzed during the current study is publicly available in the Open Science Framework (OSF) repository (https://osf.io/sw5ab/overview?view_only=60c6cff7293041c3ba21b52f26ef4eb7, accessed on 23 August 2026).

**Figure 2:**
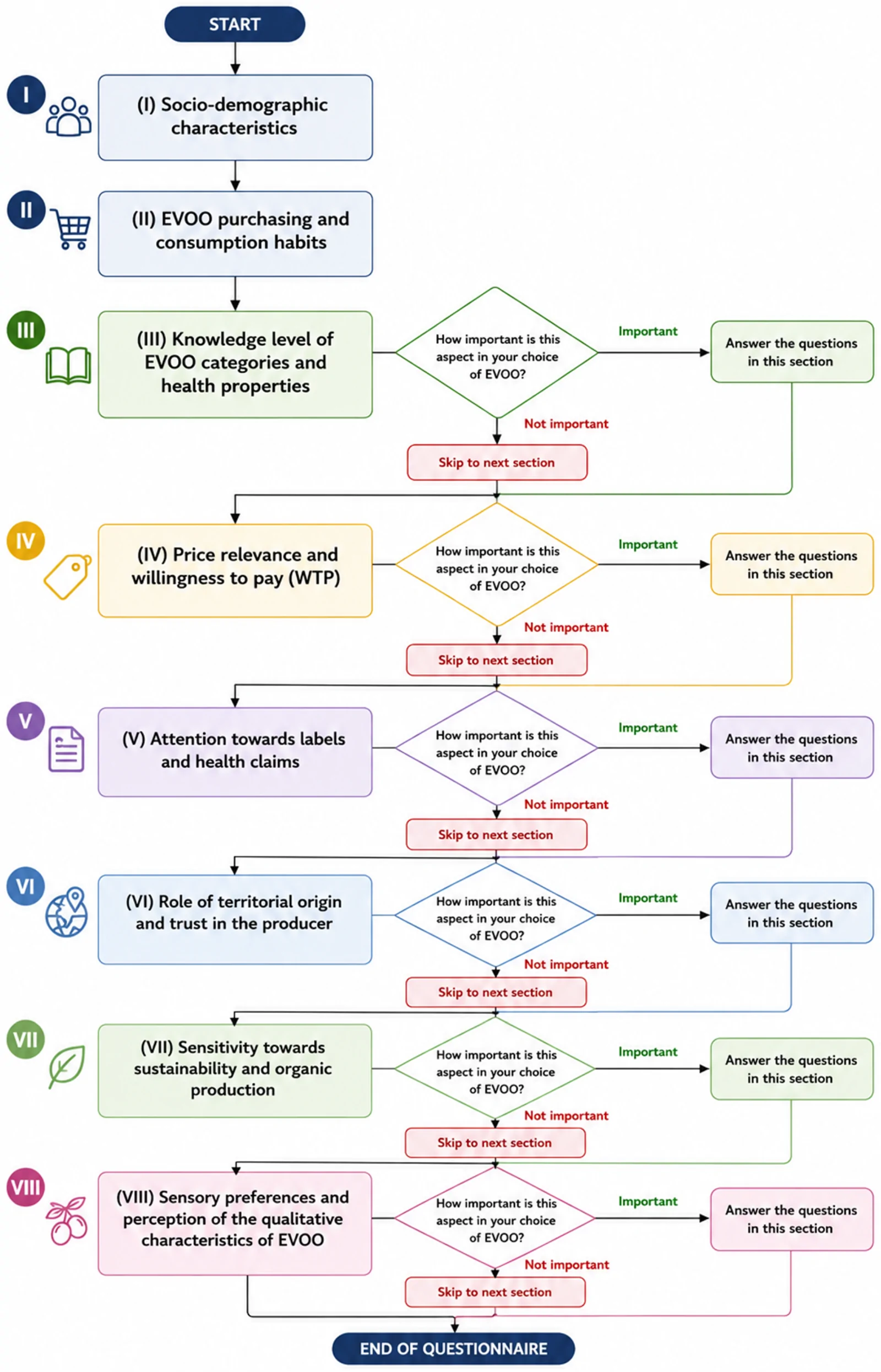
Workflow diagram of the questionnaire structure.

A total of 516 valid questionnaires were included in the analyses. Participants were recruited throughout Italy using a non-probability convenience sampling approach and through multiple recruitment channels. No commercial online recruitment company, market research agency, or pre-existing consumer panel was used. Rather, recruitment relied primarily on the researchers’ academic, professional, and personal networks. The survey was circulated among university students and academic colleagues, who represented an important component of the recruitment network, as well as among contacts outside the university setting through the researchers’ broader personal and professional networks. Recruitment was also conducted during selected in-person events, where potential participants were invited to access and complete the same online questionnaire. The use of these different channels was intended to extend recruitment beyond a single population or setting; nevertheless, participation remained entirely voluntary and based on self-selection. Consistent with these recruitment channels, university students constituted a substantial proportion of the final sample (42%), although the sample also included respondents from a broad range of occupational backgrounds, including employees, teachers and academic staff, healthcare professionals, self-employed professionals, entrepreneurs, and retirees. Respondents were subsequently grouped into three macro-regions (North, Centre, and South and Islands). For selected inferential analyses, Northern and Central respondents were combined into a single group because of the comparatively smaller sample sizes in these two macro-regions. This geographical grouping was used to examine broad regional variation in consumer behaviors and quality perceptions. Macro-region was treated as a contextual indicator rather than as a direct measure of cultural differences; accordingly, any regional variation was interpreted in relation to the broader territorial and cultural framework discussed in the literature, without assuming that geographical residence itself captures specific cultural practices, traditions, or levels of familiarity with olive oil production. Because of the voluntary recruitment procedure, the sample was not evenly distributed across geographical areas, with a higher proportion of participants from Southern Italy and the Islands. This imbalance partly reflects the recruitment context, as the research team and its academic and professional networks are primarily based in Southern Italy, where most in-person recruitment opportunities also took place. The questionnaire consisted of 44 items and included single-choice questions, multiple-choice questions, multiple-response items, and Likert-type scales. The instrument explored socio-demographic characteristics, consumption habits, perceived knowledge of EVOO, health-related beliefs, price perception, label use, territorial identity, sustainability orientation, and sensory preferences. Conditional branching was implemented so that some questions were presented only to respondents meeting predefined criteria. A graphical overview structure is reported in Figure 2.

### 2.2. Measures

#### 2.2.1. Socio-Demographic Characteristics

Socio-demographic variables included gender, age, geographical area of residence, educational level, occupation, responsibility for household food purchasing, and personal income.

#### 2.2.2. Consumption and Purchasing Behavior

Participants reported their frequency of EVOO consumption, usual place of purchase, purchase frequency, type of oil consumed, and main culinary uses.

#### 2.2.3. Health Knowledge and Health-Related Beliefs

Perceived knowledge was assessed through items investigating familiarity with different oil categories and awareness of the health benefits of EVOO. Additional questions explored beliefs regarding the reasons why EVOO is considered beneficial for health and which label elements are perceived as indicators of health-related properties.

#### 2.2.4. Price Relevance and Willingness to Pay

Economic aspects included perceived importance of price, average expenditure per liter, willingness to pay a premium for certified high-quality EVOO, acceptable price premium, and perceived fair price for such products.

#### 2.2.5. Label Use and Information Processing

Participants were asked whether they read product labels, which information they seek, how clear they perceive label information to be, and which additional information they consider useful.

#### 2.2.6. Territorial Identity and Trust

This section assessed the importance attributed to geographical indications (DOP/IGP), level of identification with the area of residence, pride in belonging to a specific territory, trust in local producers, and motivations for preferring locally produced oil.

#### 2.2.7. Sustainability and Organic Orientation

Attitudes toward sustainability were evaluated through the perceived importance of organic certification, environmentally sustainable production processes, and principles related to organic and biodynamic systems.

#### 2.2.8. Sensory Preferences and Perceived Quality Cues

Participants evaluated the importance of sensory attributes such as taste, aroma, and texture in their purchasing decisions. They also reported their preferred sensory profiles and identified the sensory characteristics they associated with EVOO quality. Both items were administered as multiple-response questions, allowing respondents to select one or more attributes among sweetness, bitterness, pungency, and fruitiness.

### 2.3. Statistical Analyses

Data were analyzed using IBM SPSS Statistics, version 29.0 (IBM Corp., Armonk, NY, USA). Descriptive statistics were computed for all variables. Categorical variables were summarized as absolute frequencies and percentages. Due to the conditional structure of the questionnaire, percentages were calculated based on valid responses for each item, and the corresponding denominators were reported when relevant. Comparative analyses across geographical areas (North, Centre, South and Islands) were conducted to identify differences in consumer behavior and perceptions. For categorical variables, Pearson’s chi-square test was applied. Effect sizes were evaluated using Cramér’s V. Ordinal variables measured on Likert-type scales were analyzed as categorical variables. Where appropriate, non-parametric tests (Kruskal–Wallis) were considered to account for their ordinal nature. For interpretative clarity, some ordinal variables were grouped into broader categories (e.g., low, medium, high levels of importance or agreement), while maintaining the original distributions in the descriptive analysis.

For multiple-response questions, each response option was recorded into a binary variable (0 = not selected; 1 = selected). Results were expressed as the proportion of respondents selecting each option, allowing for a clearer interpretation of consumer preferences and behaviors. Differences in endorsement frequencies across sensory categories were examined using Cochran’s Q test for related dichotomous variables. When Cochran’s Q tests were significant, pairwise comparisons between sensory categories were performed using McNemar tests.

Two binary logistic regression models were initially planned a priori to investigate the determinants of willingness to pay for certified EVOO and sensory misperception. However, because the vast majority of respondents expressed willingness to pay a premium for certified EVOO, the distribution of the outcome variable was highly unbalanced, resulting in unstable parameter estimates and quasi-complete separation. Consequently, inferential interpretation for willingness to pay relied on descriptive and bivariate analyses. A stable logistic regression model was retained for sensory misperception, defined as the association of sweetness with EVOO quality. Independent variables included perceived knowledge of health properties, label reading behavior, and territorial identification. All predictors were entered simultaneously using the enter method. Cases with missing values were excluded listwise. Model fit was assessed using the Hosmer–Lemeshow test, and results were reported as odds ratios (Exp(B)) with 95% confidence intervals.

## 3. Results

### 3.1. Sociodemographic Characteristics

The sample included 516 participants (61.0% female), with a mean age of 39.8 years (SD = 15.6; range: 18–83). The majority resided in Southern Italy (73%), followed by Central (16%) and Northern Italy (10%). Educational attainment was relatively high: 63% held at least a university degree. University students represented 42% of the sample, while the remaining participants reported a heterogeneous range of occupational backgrounds, including employees, teachers and academic staff, healthcare professionals, self-employed professionals, entrepreneurs, and retirees. Regarding personal annual income, 35% of respondents reported an income below €5000, 27% between €5000 and €20,000, and 26% between €20,000 and €40,000, whereas comparatively fewer respondents reported incomes above €40,000. Most participants (67%) reported being primarily responsible for household food purchases (Table 1).

### 3.2. Consumer Behavior and Purchasing Patterns

The sample was strongly characterized by the consumption of EVOO, with 94% of respondents reporting extra virgin olive oil as their primary type of oil, while other types such as virgin olive oil (4%), seed oils (1%), and uncertainty regarding oil type (1%) were only marginally represented.

**Figure 3:**
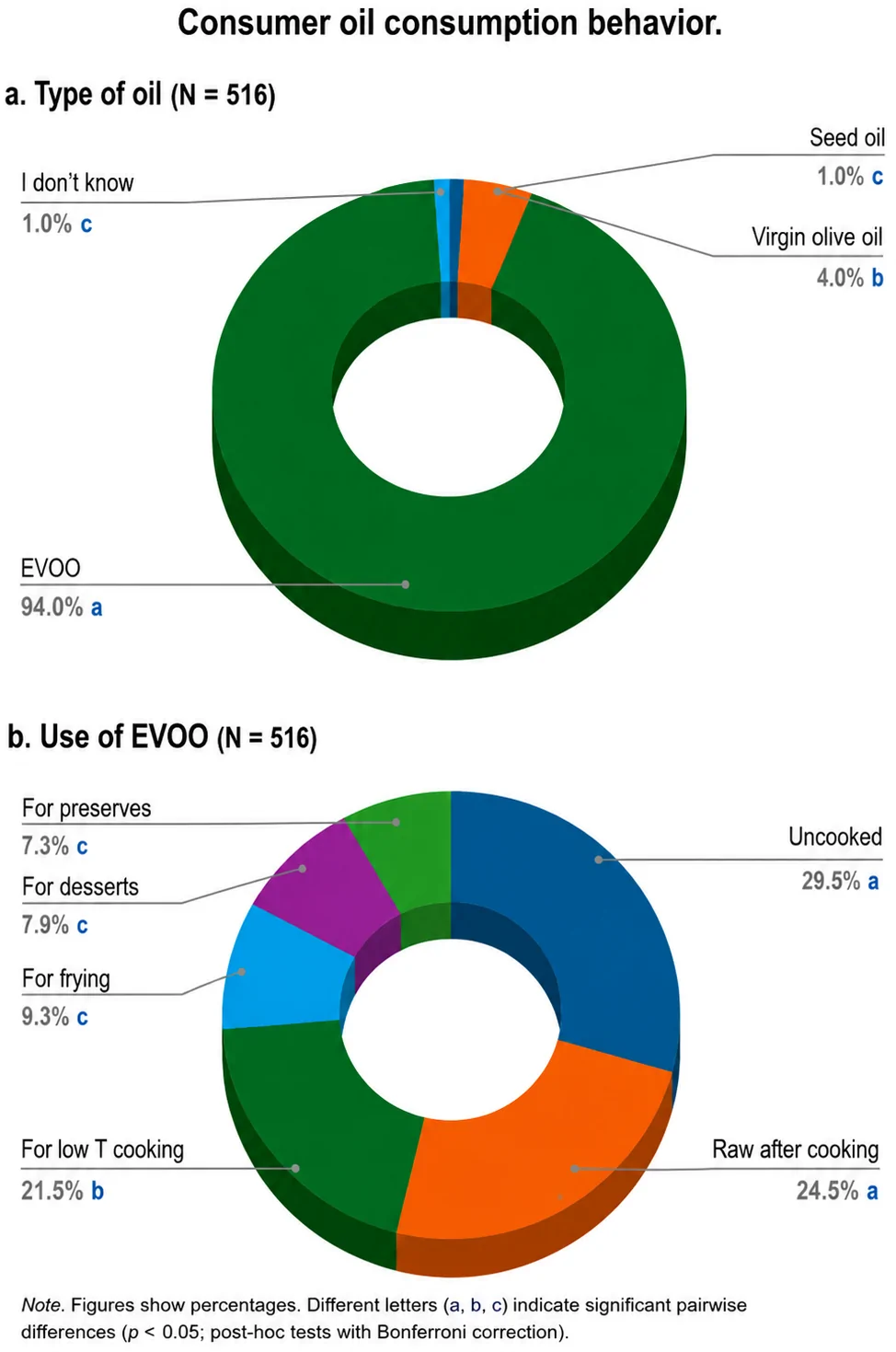
Consumer oil consumption behavior. (**a**) Type of oil mainly consumed by respondents (single-response item). (**b**) Distribution of the main uses of extra virgin olive oil (EVOO; multiple-response item); percentages represent the proportion of all reported uses accounted for by each use category. Different letters indicate statistically significant differences between response categories (*p* < 0.05).

Consumption frequency was very high, with 97% of respondents reporting frequent use. Regarding patterns of use, EVOO was most frequently consumed raw, with 91% of respondents reporting this use, corresponding to 30% of all reported uses. Other commonly reported uses included adding EVOO at the end of cooking (76% of respondents; 25% of reported uses) and cooking at low temperatures (67%; 22%), whereas frying (29%; 9%), preparation of desserts (24%; 8%), and preserves (23%; 7%) were less frequently reported (Figure 3). As multiple responses were allowed, percentages based on respondents exceed 100% when summed across categories, whereas the percentages displayed in Figure 3b represent the distribution of all reported uses.

To further examine the relationship between patterns of use and sensory quality perception, additional exploratory analyses tested whether raw use was associated with the sensory attributes considered indicators of EVOO quality. Respondents who reported using EVOO raw were less likely to associate sweetness with quality than those who did not report raw use (39% vs. 56%; χ^2^(1) = 5.30, *p* = 0.021, Cramér’s V = 0.101). Conversely, fruitiness was more frequently associated with quality among respondents reporting raw use (29% vs. 15%; χ^2^(1) = 4.39, *p* = 0.036, Cramér’s V = 0.092). Similar directional differences were observed for bitterness (31% vs. 21%) and pungency (30% vs. 17%), although these associations did not reach statistical significance (*p* = 0.133 and *p* = 0.052, respectively).

Purchase frequency was also elevated, and significant geographical differences emerged between Southern Italy and Central/Northern regions (χ^2^ = 13.825, *p* = 0.003, Cramer’s V = 0.164), with respondents from Southern Italy more likely to report higher purchasing frequency.

Regarding purchasing channels, supermarkets represented the most frequently reported source, followed by local producers, whereas mills, specialized shops, and online purchasing channels were less commonly selected. Significant geographical differences were also observed in purchasing channels (χ^2^ = 58.361, *p* < 0.001, Cramer’s V = 0.336). In particular, respondents from Southern Italy and Islands were more likely to report purchasing EVOO from local producers and mills, whereas respondents from Northern and Central regions relied more frequently on supermarkets.

### 3.3. Willingness to Pay and Price Perception

A majority of respondents declared willingness to pay a higher price for certified EVOO, with 71% indicating a positive response. Among those willing to pay a higher price, most respondents indicated a limited increase. Specifically, 51% were willing to pay up to 10% more, while 44% reported a willingness to pay between 10% and 30% more. Only a small proportion (5%) declared willingness to pay more than 30% above the base price. Interestingly, personal income was positively associated with the magnitude of the price premium respondents were willing to pay for certified EVOO (Spearman’s ρ = 0.147, *p* = 0.005, *n* = 370), indicating that respondents with higher personal income tended to report willingness to pay a larger premium. No statistically significant differences emerged between Northern/Central and Southern respondents in the proportion of individuals willing to pay a higher price (χ^2^ = 0.087, *p* = 0.768). However, statistically significant differences were found in the amount respondents were willing to pay above the base price (χ^2^ = 6.302, *p* = 0.043).

Regarding price perception, respondents most frequently identified a price range between 10 and 15 euros per liter as appropriate for a high-quality certified EVOO (45% of valid responses), followed by 5–10 euros (32%) and more than 15 euros (18%), while a smaller proportion (6%) reported uncertainty. Significant geographical differences emerged in the perceived appropriate price for certified EVOO (χ^2^ = 30.050, *p* < 0.001). In particular, respondents from Southern Italy were more frequently represented in the lower price ranges (5–10 and 10–15 euros per liter), whereas those from Northern and Central regions showed a relatively higher representation in the highest price category (above 15 euros per liter) and among respondents reporting uncertainty.

### 3.4. Health Knowledge, Beliefs, and Health-Related Quality Perception

Respondents reported moderate to high levels of perceived knowledge regarding both the classification of oils and their health properties. Concerning knowledge of differences among oil categories (e.g., extra virgin, virgin, lampante, seed oils), 28% of respondents reported a moderate level of knowledge, whereas 18% and 13% indicated high and very high levels, respectively. Lower levels of knowledge were reported by 24% and 16% of respondents. A similar pattern emerged for perceived knowledge of the health properties of extra virgin olive oil. Specifically, 27% of respondents reported a moderate level of knowledge, while 27% and 21% indicated high and very high levels, respectively. Lower levels were less frequent, with 16% and 8% reporting low or very low knowledge.

Regarding health-related beliefs, most respondents correctly associated the beneficial effects of EVOO with scientifically recognized mechanisms. Specifically, 72% agreed that EVOO is beneficial because of its content of unsaturated fatty acids, whereas 76% associated its health properties with the presence of antioxidants protecting against oxidative damage.

However, alternative explanations were also frequently endorsed. Approximately half of the sample (51%) agreed that EVOO exerts beneficial effects because its lower smoke point promotes cooking methods that preserve the nutritional properties of foods. Moreover, 74% of respondents agreed that the pleasant taste and aroma of EVOO contribute to overall well-being.

When respondents were asked which product attributes they considered associated with the health properties of EVOO, the indication of the oil category emerged as the most strongly endorsed cue, with 62% of respondents reporting high levels of perceived association. Pairwise comparisons showed that high association responses were significantly more frequent than moderate and low association responses, whereas moderate responses were also more frequent than low responses.

Similarly, nutritional information (61%), BIO certification (58%), and DOP/IGP certification (58%) were frequently perceived as indicators of health-related quality. For nutritional information, BIO certification, and DOP/IGP certification, high association responses were significantly more frequent than both moderate and low responses, whereas no significant differences emerged between moderate and low association responses.

More than half of the respondents also reported high levels of association for physicochemical properties (55%), whereas lower proportions reported moderate (25%) or low levels of association (20%). Pairwise comparisons indicated that high association responses were significantly more frequent than both moderate and low responses, while moderate and low responses did not differ significantly.

In contrast, price per liter represented the least health-relevant cue. Only 30% of respondents reported high levels of association between price and health properties, whereas 44% reported low levels of association. Low association responses were significantly more frequent than both moderate and high association responses, whereas moderate and high association responses did not differ significantly.

**Figure 4:**
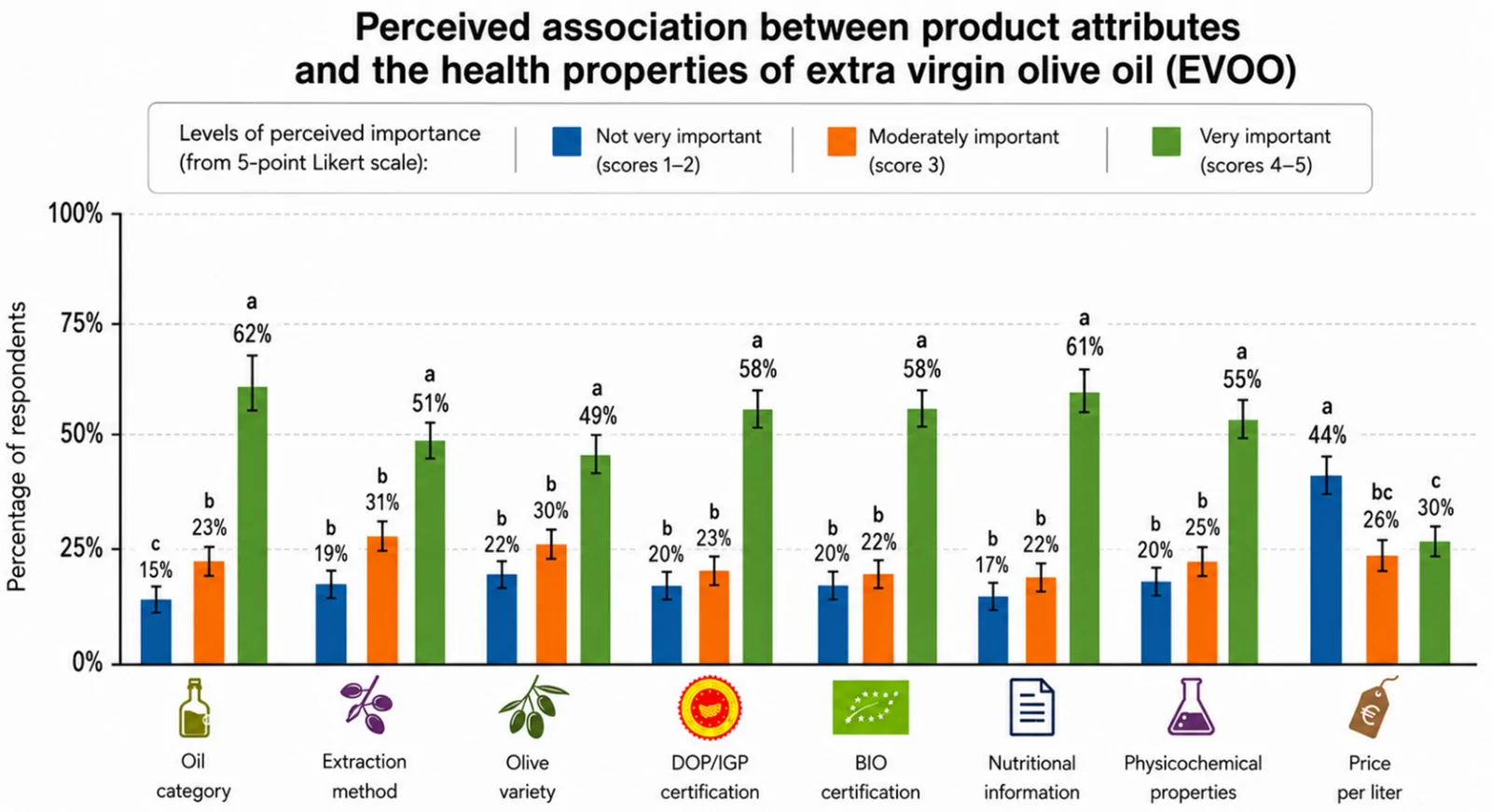
Perceived association between different product attributes and the health properties of extra virgin olive oil (EVOO). Bars represent the percentage of responses across three levels of perceived importance, derived from the original 5-point Likert scale: “Not very important” (scores 1–2), “Moderately important” (score 3), and “Very important” (scores 4–5). Error bars represent standard errors of the proportions. Different letters indicate statistically significant differences among response categories within each product attribute according to pairwise chi-square comparisons (*p* < 0.05); categories sharing the same letter do not differ significantly.

Overall, these findings suggest that respondents attributed greater health relevance to technical and compositional information than to economic cues (Figure 4).

### 3.5. Territorial Identity and Sensory Quality Cues

Beyond health-related beliefs, respondents also attributed importance to territorial and relational dimensions of EVOO quality. Significant geographical differences emerged in the importance assigned to DOP/IGP certifications referring to the area of origin (χ^2^ = 13.551, *p* = 0.004), with respondents from Southern Italy showing greater sensitivity to origin-based quality cues.

Variables reflecting attachment to the territory showed consistently high values across the sample. High levels of geographical identification (scores 4–5) were reported by 74% of respondents, whereas 75% expressed high levels of pride in belonging to their geographical area. Both variables showed significant geographical differences (identification: χ^2^ = 16.588, *p* = 0.002; territorial pride: χ^2^ = 16.091, *p* = 0.003), with higher levels observed among respondents from Southern Italy. Trust in local producers was also widespread, with 76% of respondents reporting high levels of trust (scores 4–5). However, no significant geographical differences emerged for this variable (χ^2^ = 7.526, *p* = 0.111), suggesting that trust in producers is consistently high across regions.

**Figure 5:**
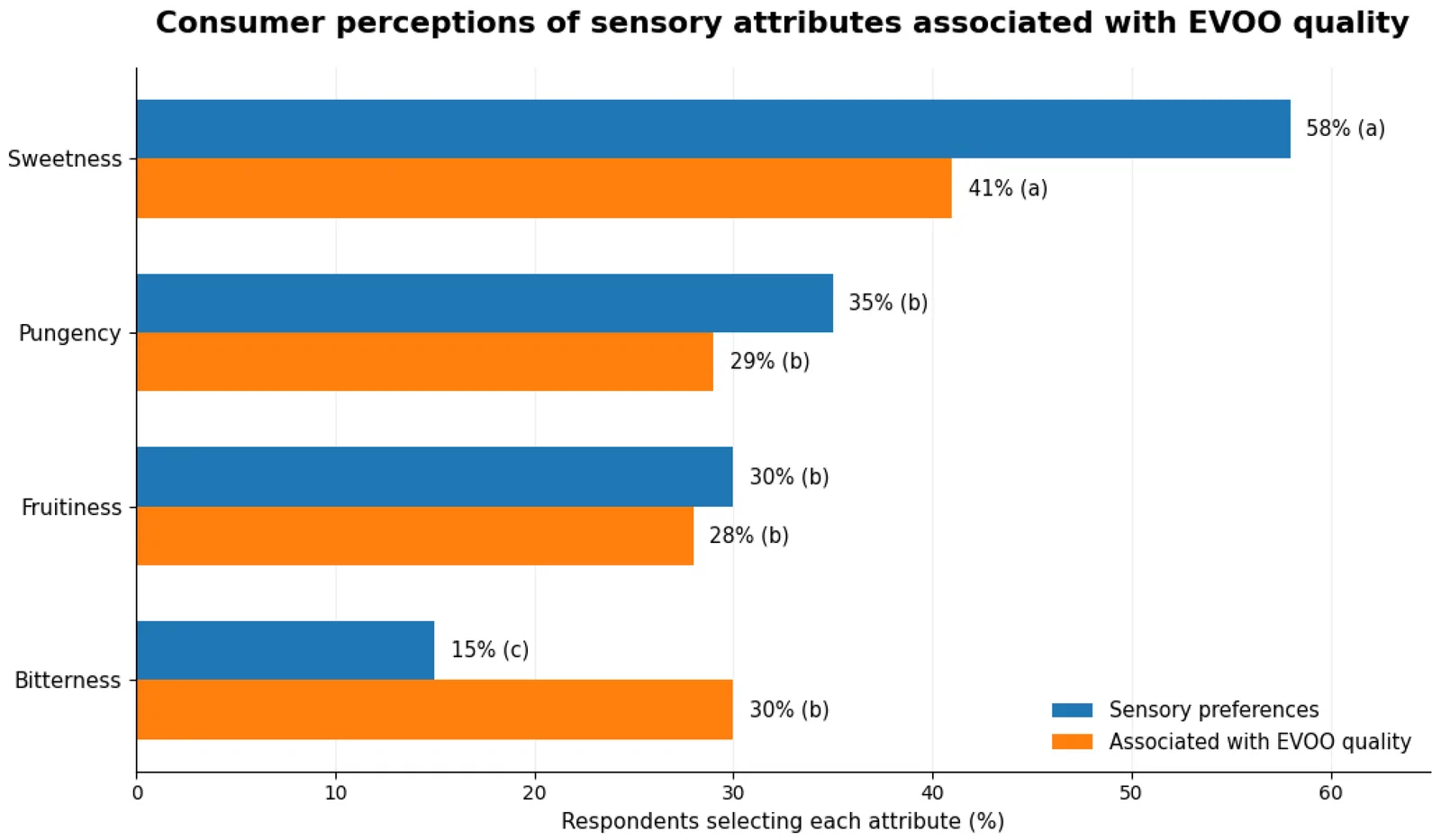
Consumers’ sensory preferences and sensory attributes associated with perceived extra virgin olive oil (EVOO) quality. Bars represent the percentage of respondents selecting each sensory attribute as a preferred sensory characteristic or as an indicator of EVOO quality. Multiple responses were allowed. Different letters indicate statistically significant differences among sensory attributes within each question according to pairwise McNemar comparisons (*p* < 0.05); categories sharing the same letter do not differ significantly.

With respect to sensory preferences, respondents showed heterogeneous taste profiles (Figure 5). Sweetness emerged as the most frequently preferred sensory characteristic, being selected by 58% of participants. Pungency and fruitiness were preferred by 35% and 30% of respondents, respectively, whereas bitterness was selected less frequently (15%). The distribution of preferences differed significantly across sensory categories (Cochran’s Q = 182.74, df = 3, *p* < 0.001), indicating that consumers did not equally endorse the four sensory profiles. Pairwise comparisons further showed that sweetness was selected significantly more frequently than bitterness, pungency, and fruitiness (all McNemar tests, *p* < 0.001). Moreover, both pungency and fruitiness were preferred significantly more often than bitterness (both *p* < 0.001), whereas no significant difference emerged between pungency and fruitiness (*p* = 0.146).

When respondents were asked which sensory characteristics they associated with EVOO quality, sweetness was once again the most frequently endorsed attribute (41%), followed by bitterness (30%), pungency (29%), and fruitiness (28%). The distribution of responses also differed significantly across categories (Cochran’s Q = 22.52, df = 3, *p* < 0.001). Pairwise comparisons indicated that sweetness was associated with quality significantly more often than bitterness (*p* = 0.002), pungency (*p* = 0.001), and fruitiness (*p* < 0.001), whereas no significant differences emerged among bitterness, pungency, and fruitiness (all *p* > 0.05).

### 3.6. Determinants of Sensory Misperception

Based on the sensory patterns described above, the association between sweetness and EVOO quality was used as an indicator of sensory misperception, operationally defined in the present study as the endorsement of sweetness as a sensory indicator of EVOO quality. This operational definition refers specifically to a quality-related sensory belief and does not imply that a preference for sweetness is itself incorrect or that sweetness is necessarily perceived as an indicator of healthiness. A multivariable binary logistic regression model was conducted to investigate the determinants of sensory misperception (Table 2). The model was statistically significant, χ^2^(3) = 16.11, *p* = 0.001, but its explanatory power was limited (Nagelkerke R^2^ = 0.059), indicating that the predictors included in the model accounted for only a small proportion of the variability in sensory misperception. Accordingly, the model should be interpreted as an exploratory analysis of associations rather than as a predictive model. Nevertheless, the model showed adequate calibration according to the Hosmer–Lemeshow test (χ^2^ = 4.82, *p* = 0.777).

Perceived knowledge of EVOO health properties emerged as the only significant predictor, with higher levels of knowledge associated with a lower probability of associating sweetness with EVOO quality (OR = 0.713, 95% CI = 0.573–0.888, *p* = 0.002). In contrast, label reading behavior and territorial identification were not significant predictors.

These results suggest that sensory misperception is partly associated with knowledge-related factors, whereas label reading behavior and territorial identification did not show significant associations in this model.

## 4. Discussion

The present study provides an integrated perspective on consumer behavior, quality perception, and sensory misperception in the context of EVOO. The findings reveal that high product involvement and strong quality orientation may coexist with persistent misperceptions regarding key quality indicators. By simultaneously considering health-related knowledge, territorial identity, and sensory beliefs, the study highlights how consumers construct the perceived value of EVOO through the interplay of cognitive, symbolic, and sensory factors.

A first key insight concerns the profile of EVOO consumers emerging from the data. The sample is characterized by very frequent consumption and regular purchasing behavior, confirming the centrality of EVOO within everyday dietary practices. More specifically, EVOO was by far the predominant type of oil consumed, and its widespread use in uncooked applications further indicates that it represents an integral component of respondents’ everyday dietary practices. This result is consistent with previous research highlighting the cultural and nutritional relevance of EVOO in Mediterranean countries [1,2]. At the same time, consumer behavior appears to reflect a dual orientation. On the one hand, respondents show a clear preference for quality, as evidenced by the high proportion of individuals willing to pay a premium for certified EVOO and the importance attributed to origin-related attributes. On the other hand, purchasing patterns reveal the coexistence of modern and traditional channels, with large-scale retail distribution playing a significant role alongside direct purchasing from producers and local mills. This pattern suggests that consumers seek both convenience and authenticity, combining modern purchasing practices with more traditional consumption habits. Geographical differences provide further insight into this pattern. Respondents from Southern Italy reported more frequent purchasing from local producers and mills and attributed greater importance to territorial identification and origin-based quality cues. These findings are consistent with previous evidence showing that geographical origin, local production, and regional identity can play an important role in consumers’ evaluation of EVOO [25,27,28]. One possible interpretation is that regional differences in purchasing practices and quality perceptions are embedded within different degrees of familiarity with local production systems and food traditions. This interpretation may be particularly relevant in areas where olive cultivation and direct relationships with producers constitute a more visible component of the local food environment. However, geographical residence should not be interpreted as a direct measure of culture or familiarity with olive production. The observed regional differences therefore indicate contextual variation in consumer practices and perceptions, while the specific cultural, historical, and experiential mechanisms underlying such variation remain to be directly investigated. However, willingness to pay a premium does not differ significantly across geographical areas, indicating that quality orientation is a widespread characteristic rather than a territorially segmented one. This suggests that while the symbolic meaning of quality may vary across regions, its perceived importance is broadly shared.

A second important contribution of the study concerns consumers’ quality perception. The findings suggest that consumers recognize some scientifically relevant cues, but simultaneously integrate them with symbolic and heuristic cues, generating a multidimensional and only partially accurate representation of EVOO quality. Consumers appear to evaluate EVOO through a combination of informational cues, territorial attributes, trust in producers, and sensory expectations rather than exclusively through objective product characteristics. In this sense, quality perception extends beyond purely chemical or nutritional properties and incorporates relational and experiential dimensions [29].

This interpretation is consistent with previous research showing that quality evaluation in traditional food products is influenced not only by objective characteristics but also by symbolic meanings, territorial identity, and trust-related mechanisms. In the context of EVOO, geographical indications such as DOP and IGP represent important quality signals for consumers, while trust in producers and attachment to the territory contribute to reducing uncertainty and enhancing perceived authenticity [25,27,28]. At the same time, the findings reveal that consumers also incorporate non-scientific cues into their evaluations. Although respondents correctly recognized the importance of antioxidants, unsaturated fatty acids, and several health-related attributes, they also incorporated sensory expectations and simplified heuristics into their evaluations. As a result, quality perception appears to reflect the coexistence of accurate and inaccurate cues rather than a fully coherent understanding of product characteristics. Overall, these findings suggest that quality perception cannot be understood solely in terms of objective attributes, but should be interpreted within a socio-cultural framework. This reflects a broader issue discussed in the literature, namely that increasing the amount of information available to consumers does not necessarily translate into better understanding [30]. Rather than processing all available information in an integrated manner, consumers often rely on simplified decision rules and focus on a limited number of salient cues. These findings are particularly relevant in the context of EFSA-approved health claims for EVOO polyphenols. Although scientific evidence and European regulations clearly identify specific compounds and nutritional properties associated with health benefits, consumers appear to interpret health-related information in a partial and sometimes inconsistent way. This discrepancy may contribute to the still limited use and effectiveness of EFSA health claims in EVOO communication strategies, despite their potential role in supporting informed consumer choices. In other words, the availability of scientifically validated information does not automatically guarantee its correct interpretation by consumers. Interestingly, this pattern also emerged with respect to price perception. Although 71% of respondents declared a willingness to pay a premium for certified EVOO, price itself was rarely interpreted as a health-related cue. Specifically, only 30% considered price per liter strongly associated with health properties, whereas 44% assigned low levels of association to this attribute. These findings suggest that consumers distinguish between economic value and perceived health value. Consumers appear willing to spend more for quality, but do not necessarily interpret higher prices as indicators of superior health-related characteristics.

Previous studies have shown that consumers are willing to pay a premium for specific EVOO quality attributes, particularly origin-related characteristics and innovative production methods [23]. However, the relationship between price and perceived quality is not always linear. Consumers process multiple quality signals simultaneously, and willingness to pay is influenced by a combination of functional, symbolic, and relational dimensions rather than by price alone [22]. The present findings extend this evidence by showing that higher economic value is not automatically translated into higher perceived health value. This result further supports the idea that quality perception is multidimensional and influenced by symbolic and experiential dimensions rather than by a simple price–quality heuristic.

This interpretation is further supported by the heterogeneity observed in the perceived relevance of label information for health-related properties. Nutritional information is generally recognized as important, but consumers appear to attribute different levels of health relevance to the various product attributes and information cues considered. This suggests that consumers do not process label information as an integrated system, but rather as a collection of loosely connected cues whose meaning is not always fully understood.

One of the most relevant findings of the present study concerns the mismatch between scientifically recognized sensory indicators of EVOO quality and consumers’ perceptions. Although bitterness and pungency are closely associated with the presence of phenolic compounds and are generally considered markers of high-quality EVOO, sweetness emerged as the sensory attribute most frequently associated with quality (41%). Bitterness, pungency, and fruitiness were selected by 30%, 29%, and 28% of respondents, respectively.

These findings reveal a discrepancy between consumer beliefs and scientifically recognized indicators of EVOO quality. Importantly, the present results do not imply that sweeter sensory profiles are inherently undesirable. Rather, they suggest that sweetness is not considered, within expert sensory evaluation, a primary indicator of phenolic richness or a recognized positive sensory attribute of EVOO. Because bitterness and pungency are closely linked to the presence of bioactive phenolic compounds, consumers who associate quality mainly with milder or sweeter sensory experiences may rely on criteria that only partially overlap with objective quality standards. Previous studies have similarly shown that consumers often perceive bitterness as an undesirable characteristic and tend to prefer milder sensory profiles, despite the association between these sensory attributes and the presence of bioactive compounds [12,15,17,18].

This pattern suggests that sensory evaluation is strongly influenced by expectations, habits, and prior experiences rather than by formal knowledge alone [26]. Consumers do not reject quality per se; rather, they interpret sensory signals through simplified and sometimes misleading heuristics. As a consequence, incorrect associations—such as linking sweetness with superior quality—can persist even among frequent consumers. The additional analyses on patterns of use provide further context for interpreting these sensory associations. Raw consumption was highly prevalent in the sample, with 91% of respondents reporting using EVOO in this way. Importantly, respondents who used EVOO raw were less likely to associate sweetness with quality and more likely to identify fruitiness as a quality attribute. Bitterness and pungency showed similar directional patterns, although these differences did not reach statistical significance. These findings suggest that patterns of use may be related to how consumers interpret sensory quality cues. In particular, consuming EVOO raw may provide greater opportunity for direct exposure to its sensory characteristics than uses involving cooking or food preparation. However, given the exploratory and cross-sectional nature of these analyses, this interpretation should be considered tentative and does not establish that raw consumption leads to greater sensory knowledge.

Beyond these exploratory associations with patterns of use, the regression analysis provides further insight into the role of knowledge-related factors in sensory misperception. Perceived knowledge of EVOO health properties emerged as the only significant predictor included in the model, with higher levels of perceived knowledge associated with a lower probability of associating sweetness with quality. In contrast, label reading behavior and territorial identification were not significantly associated with sensory misperception. However, the limited explanatory power of the model indicates that these variables capture only a small proportion of the variability in sensory misperception. The findings should therefore not be interpreted as identifying the primary determinants of sensory misperception, but rather as evidence of a specific association between perceived knowledge and the likelihood of endorsing sweetness as a quality attribute. Other individual, experiential, contextual, and sensory factors not assessed in the present study are likely to contribute to these perceptions and should be investigated in future research.

**Figure 6:**
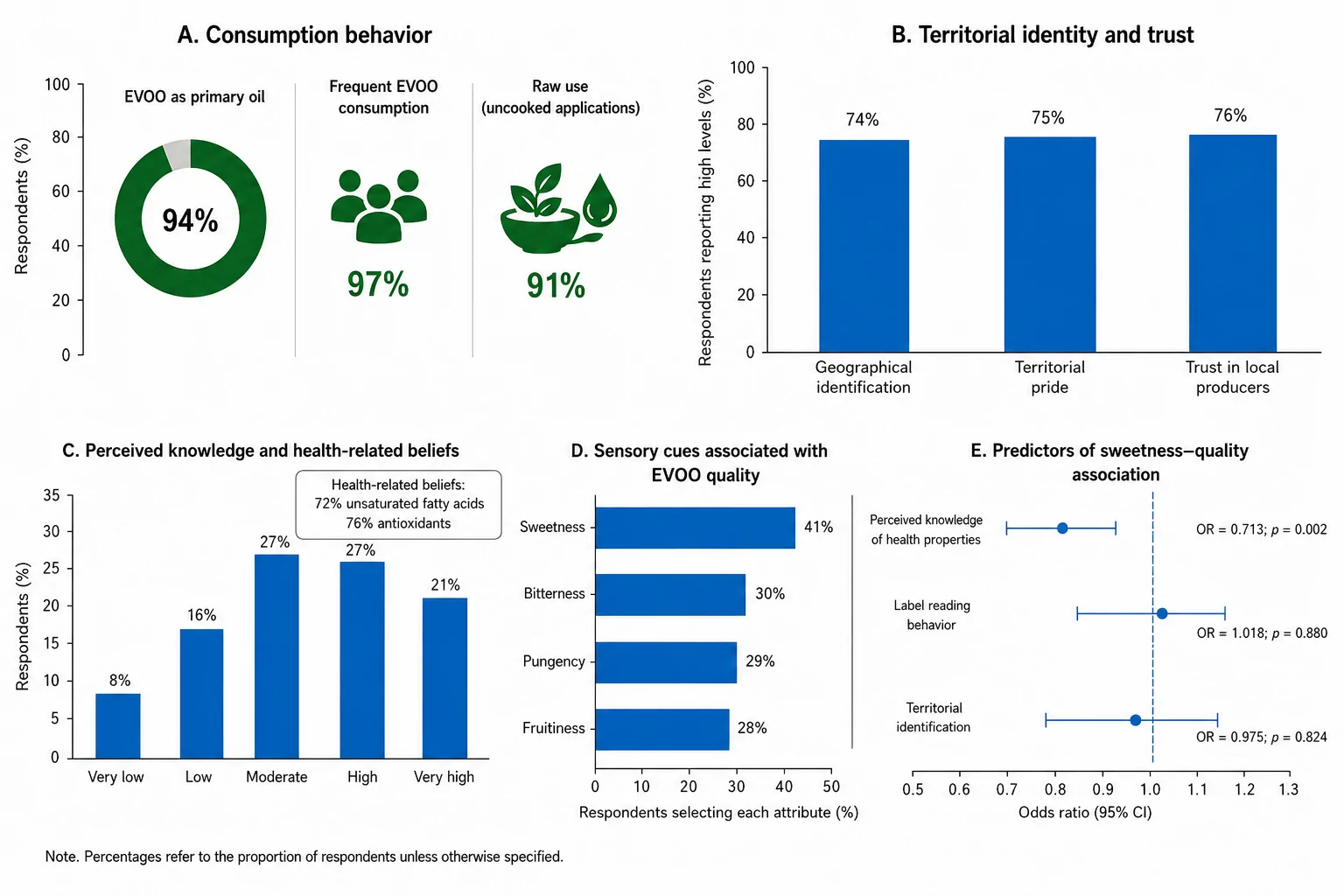
Summary of the main findings concerning consumer perceptions of extra virgin olive oil (EVOO) quality. (**A**) Main consumption patterns, including EVOO as the primary type of oil consumed (94%), frequent EVOO consumption (97%), and raw use (91%). (**B**) Overall levels of geographical identification, territorial pride, and trust in local producers. (**C**) Distribution of perceived knowledge of EVOO health properties and endorsement of selected health-related beliefs. (**D**) Sensory attributes associated with perceived EVOO quality; percentages represent the proportion of respondents selecting each attribute, and multiple responses were allowed. (**E**) Multivariable logistic regression predicting the association of sweetness with EVOO quality. Points represent odds ratios (ORs), horizontal bars indicate 95% confidence intervals, and the dashed vertical line indicates OR = 1. Higher perceived knowledge of EVOO health properties was associated with lower odds of identifying sweetness as an indicator of EVOO quality (OR = 0.713, 95% CI: 0.573–0.888, *p* = 0.002), whereas label-reading behavior and territorial identification were not significant predictors.

Taken together, these findings illustrate how informational, territorial, and sensory dimensions coexist in consumers’ evaluations of EVOO quality. As summarized in Figure 6, consumers showed substantial engagement with scientifically relevant health and quality information, while simultaneously relying on territorial cues and sensory associations that did not always correspond to established indicators of EVOO quality.

Overall, the results point to sensory education and experiential learning as potentially relevant avenues for addressing the gap between consumer perceptions and scientifically recognized quality criteria. In particular, interventions aimed at improving consumers’ understanding of the relationship between phenolic compounds, health properties, and sensory attributes warrant further investigation as possible strategies for enhancing the effectiveness of quality communication. These findings may also be interpreted within the framework of affect–cognition matching in persuasion, which proposes that persuasive messages are more effective when their content is aligned with the dominant basis on which individuals evaluate an object [31]. In the context of EVOO, institutional and health-related communication typically relies on cognitive information, such as nutritional claims, chemical composition, and certification schemes. However, consumers often evaluate EVOO through affective and sensory dimensions, including taste, familiarity, trust, and attachment to local traditions. When communication strategies are not aligned with the way consumers evaluate a product, scientifically accurate information may have a limited impact on consumer judgments and behavior. From this perspective, the present findings are consistent with the possibility that increasing the amount of available information alone may not necessarily improve consumer understanding. Rather, future research should examine whether the effectiveness of EVOO communication depends on how information is framed and on its correspondence with consumers’ existing beliefs and evaluative processes. In particular, affective and symbolic dimensions—such as territorial identity and trust—may represent relevant contextual factors to consider when investigating how consumers respond to quality-related communication, alongside more cognitively oriented information concerning nutritional properties, health claims, and product composition.

From a broader perspective, the findings suggest that a purely informational approach may not fully capture the complexity of consumer evaluations of EVOO. Respondents appeared to rely on a combination of cognitive, symbolic, and sensory cues, indicating that future research on quality communication should consider how these different dimensions interact. In particular, experimental studies could examine whether integrating scientifically grounded information with sensory and experiential elements improves consumers’ understanding of EVOO quality compared with information-based communication alone.

From an applied perspective, the present findings identify several directions that may be relevant for future communication and consumer-education initiatives, although their effectiveness cannot be established from the present survey. The coexistence of scientifically grounded knowledge with partially inaccurate sensory quality beliefs suggests that the availability of certifications and health-related information does not necessarily ensure consistent interpretation of EVOO quality cues. Future intervention studies could therefore test whether simplified health-related information, sensory education, comparative tasting, or appropriately contextualized information about origin and production improve consumers’ understanding of these cues. Such evidence would be necessary before drawing specific policy or managerial recommendations regarding the most effective strategies for EVOO quality communication [32].

### Study Limitations and Future Research

Several limitations of the present study should be acknowledged and provide directions for future research. First, participants were recruited through a non-probability convenience sampling strategy based primarily on the researchers’ academic, professional, and personal networks, complemented by recruitment during selected in-person events. Participation was therefore based on self-selection, and the resulting sample should not be considered representative of the Italian consumer population. This recruitment strategy also contributed to a substantial proportion of university students in the sample (42%) and to a relatively highly educated participant profile. These characteristics may have influenced reported levels of perceived knowledge, attention to product information, and other quality-related evaluations. Future studies should therefore replicate the present findings using probability-based or stratified sampling procedures and samples that more closely reflect the demographic, educational, occupational, and socioeconomic composition of the Italian population.

Second, the geographical distribution of the sample was markedly unbalanced, with respondents from Southern Italy and the Islands accounting for the majority of participants. Consequently, comparisons across macro-regions should be interpreted cautiously, particularly because the smaller Northern and Central subsamples provide less precise estimates of regional patterns. Moreover, geographical residence was used as a broad contextual indicator and should not be interpreted as a direct measure of cultural differences, local food traditions, or familiarity with olive production. Although the observed regional patterns may be interpreted in light of previous literature on territorial identity and local production systems, the present design cannot identify the cultural or historical mechanisms underlying these differences. Future research should recruit more geographically balanced samples and directly assess potentially relevant contextual factors, including familiarity with olive cultivation, exposure to local production systems, regional food traditions, and previous experience with locally produced EVOO.

Third, the cross-sectional and correlational nature of the study precludes causal inference. The associations observed between perceived knowledge, information-related behaviors, territorial factors, and sensory quality beliefs do not establish the direction of these relationships. This consideration is particularly important for the association between perceived knowledge of EVOO health properties and a lower likelihood of identifying sweetness as an indicator of quality. Longitudinal and experimental studies could examine whether increasing consumer knowledge or exposure to specific types of information actually produces changes in sensory quality beliefs. Experimental manipulation of health-related information, labeling formats, or sensory education would also allow stronger conclusions regarding the effectiveness of different communication strategies.

Fourth, knowledge was assessed primarily through self-reported perceptions rather than through an objective test of consumers’ factual knowledge. Perceived knowledge is theoretically relevant because consumers’ confidence in what they know may itself influence judgments and choices; however, it should not be equated with objectively accurate knowledge. Future studies should therefore assess subjective and objective knowledge simultaneously to determine whether they have distinct or interactive relationships with sensory beliefs, label interpretation, and willingness to pay.

Fifth, sensory quality perception was assessed through self-reported associations rather than through controlled sensory evaluation of actual EVOO samples. The designation of sweetness as a sensory misperception in the present study refers specifically to its endorsement as an indicator of EVOO quality and should not be interpreted as implying that a personal preference for sweeter or milder oils is itself incorrect. Furthermore, without blind tasting or chemical characterization of oils, the present data cannot directly compare consumers’ sensory judgments with the measured phenolic composition or sensory profile of specific products. Future research combining questionnaires with blind tasting sessions, trained sensory evaluation, and chemical characterization could provide a more direct test of the correspondence between perceived and objectively assessed quality attributes.

Finally, although the logistic regression model identified a significant association between perceived knowledge and sensory misperception, its explanatory power was limited. The predictors included in the model accounted for only a small proportion of the variability in the outcome, indicating that sensory quality beliefs are likely shaped by additional factors not captured in the present study. Future research should consider a broader range of individual and contextual determinants, including sensory experience, frequency and modality of EVOO use, familiarity with different sensory profiles, food involvement, health orientation, previous exposure to sensory education, and characteristics of the purchasing environment. The exploratory association observed in the present study between raw EVOO consumption and sensory quality beliefs may provide one starting point for investigating these experiential factors.

More broadly, future research would benefit from integrating survey, experimental, sensory, and behavioral approaches. Such designs could examine whether interventions based on simplified labeling, comparative tasting, sensory education, or communication linking phenolic compounds to bitterness and pungency improve consumers’ ability to interpret EVOO quality cues. Cross-cultural and cross-regional studies using comparable measures would also help determine which patterns are specific to the Italian context and which reflect more general mechanisms of EVOO evaluation. Together, these approaches could clarify how objective knowledge, subjective knowledge, sensory experience, and territorial context interact in shaping consumer judgments and provide a stronger empirical basis for designing effective quality and health communication strategies.

## 5. Conclusions

This study provides evidence on how Italian consumers perceive and evaluate EVOO quality, highlighting the coexistence of high product involvement with heterogeneous understandings of health-related and sensory quality cues. Within the present sample, consumer evaluations reflected informational, territorial, and sensory dimensions, while some perceived quality cues did not fully correspond to scientifically recognized characteristics of EVOO. In particular, sweetness was frequently associated with quality, and higher perceived knowledge of EVOO health properties was associated with a lower likelihood of endorsing this association. However, the limited explanatory power of the regression model indicates that perceived knowledge accounts for only a small part of the variability in sensory quality beliefs and that additional individual, experiential, and contextual factors are likely to be involved. The findings also suggest that geographical origin, territorial identification, and trust in local producers are relevant components of the broader context in which consumers evaluate EVOO. Nevertheless, given the cross-sectional design, non-probability sampling procedure, and geographical imbalance of the sample, these patterns should not be interpreted as evidence of causal relationships or generalized to the Italian population as a whole. Rather, they identify associations and consumer-perception patterns that warrant further investigation in more representative samples and through experimental and longitudinal designs.

From an applied perspective, the observed discrepancies between scientifically established characteristics and consumer perceptions suggest that simply increasing the amount of technical information available to consumers may not necessarily ensure accurate understanding. Future experimental research should evaluate whether approaches combining clear health-related information with sensory education, tasting experiences, or appropriately contextualized information about origin and production can improve consumers’ interpretation of EVOO quality cues. Such research would help establish which communication strategies, if any, are effective in reducing the gap between perceived and scientifically established indicators of EVOO quality.

## Figures and Tables

**Table 1:** Sociodemographic characteristics of the sample (*N* = 516).

Variable	Category	n	%
Age (years)	Mean (SD)	39.8 (15.6)	—
	Range	18–83	—
Gender	Female	315	61
	Male	199	39
	Other/Prefer not to say	2	<1
Geographical area	Southern Italy and Islands	379	73
	Central Italy	84	16
	Northern Italy	53	10
Education level	Lower secondary or below	21	4
	Upper secondary	169	33
	Bachelor’s degree	112	22
	Master’s degree	162	31
	Postgraduate/PhD	52	10
Current occupational status	University student	214	42
	Other occupational status	275	53
	Not reported	27	5
Personal annual income (€)	<5000	179	35
	5000–20,000	138	27
	20,000–40,000	135	26
	40,000–60,000	38	7
	>60,000	25	5
	Missing	1	<1
Responsibility for household food purchases	Yes	348	67
	Shared	128	25
	No	40	8

Note: Percentages may not sum to 100% due to rounding.

**Table 2:** Binary logistic regression model predicting sensory misperception, operationalized as the association between sweetness and EVOO quality.

Predictor	B	SE	OR (95% CI)	*p*
Perceived knowledge of EVOO health properties	−0.338	0.111	0.713 (0.573–0.888)	0.002
Label reading behavior	0.018	0.119	1.018 (0.807–1.284)	0.880
Territorial identification	−0.025	0.112	0.975 (0.783–1.215)	0.824

Model statistics: χ^2^(3) = 16.11, *p* = 0.001; Nagelkerke R^2^ = 0.059; Hosmer–Lemeshow χ^2^(8) = 4.82, *p* = 0.777. Note. OR = odds ratio; CI = confidence interval. Odds ratios below 1 indicate a lower probability of associating sweetness with EVOO quality.

## Data Availability

The dataset generated and analyzed during the current study is publicly available in the Open Science Framework (OSF) repository at https://osf.io/sw5ab/overview?view_only=60c6cff7293041c3ba21b52f26ef4eb7 (accessed on 23 August 2026).
